# Neoadjuvant PD-1 blockade induces the autophagy of immune cells: a new target for synergistic therapy of recurrent glioblastoma

**DOI:** 10.1016/j.bbrep.2025.102119

**Published:** 2025-06-27

**Authors:** Zixue Xuan, Kai Wang, Qingxia Zhu, Ting Sun, Jinying Jiang, Zhongxiu Wu, Shuilian Zheng, Hongying Zhao

**Affiliations:** a Department of Pharmacy, Zhejiang Provincial People's Hospital Bijie Hospital, Bijie, Guizhou, China; b Center for Clinical Pharmacy, Cancer Center, Department of Pharmacy, Zhejiang Provincial People's Hospital (Affiliated People's Hospital), Hangzhou Medical College, Hangzhou, Zhejiang, China; c Key Laboratory of Epigenetics and Oncology, Research Center for Preclinical Medicine, Southwest Medical University, Luzhou, China; d Department of Pharmacy, Shanghai Ninth People’s Hospital, Shanghai JiaoTong University School of Medicine, Shanghai, 200199, China; e Department of Pathology, The First Affiliated Hospital, School of Medicine, Zhejiang University, Hangzhou, China

**Keywords:** Glioblastoma, Neoadjuvant, Immunotherapy, Autophagy, Cell heterogeneity

## Abstract

**Background:**

Neoadjuvant PD-1 blockade may incidentally modulate autophagy in immune cells, which could contribute to drug resistance and tumor relapse. However, the specific immune cell subsets affected by neoadjuvant PD-1 blockade in terms of autophagy remain to be fully elucidated, as well as the drugs that might influence these processes.

**Methods:**

Single-cell sequencing data from tissues of recurrent glioblastoma (GBM.rec) and GBM treated with neoadjuvant PD-1 blockade (GBM.PD1) were analyzed to investigate the changes in autophagy within immune cells in the GBM.PD1 group. Subsequently, the functional characteristics of subtypes regulated by membrane proteins were explored, and potential drugs targeting key immune cell subsets mediated by these proteins were identified.

**Results:**

Neoadjuvant PD-1 blockade significantly increased the proportion of lymphoid cells with elevated autophagy. This elevated autophagy level was associated with specific ligand-receptor interactions in GBM, such as HLA-DRA–CD4. Immune cell subtypes, particularly those with both lymphoid and myeloid signatures (L + M cells, APOE + cells), exhibited strong associations with autophagy. These L + M cells demonstrated significantly more T cell-related interactions in the GBM.PD1 group, with notable receptor-ligand interactions like GZMA-F2R. Furthermore, ribavirin, which targets CXCL8 and IL6, was identified as a potential drug candidate for targeting L + M cells.

**Conclusion:**

L + M cells may represent critical immune components involved in autophagy induced by neoadjuvant PD-1 blockade. The interactions between HLA-DRA and CD4, as well as between GZMA and F2R, are crucial for modulating immune responses. Moreover, ribavirin, targeting CXCL8 and IL6, has the potential to enhance the efficacy of neoadjuvant PD-1 blockade.

## Highlights

• L + M cells may be critical immune components involved in autophagy induced by neoadjuvant PD-1 blockade.
• Interactions between HLA-DRA and CD4, as well as GZMA and F2R, are crucial for modulating immune responses.
• Ribavirin, targeting CXCL8 and IL6, could potentially enhance the efficacy of neoadjuvant PD-1 blockade.

## 1 Introduction

Glioblastoma (GBM) is characterized by rapid proliferation, high recurrence rates, and a grim prognosis [1]. Despite the remarkable efficacy of current immunotherapies against solid tumors, their application in GBM treatment faces significant challenges [2]. For example, targeting the programmed cell death protein 1 (PD-1)/programmed death-ligand 1 (PD-L1) signaling pathway has not improved survival rates for GBM patients [[2], [3], [4]]. However, emerging evidence suggests that PD-1 blockade may reshape the tumor immune microenvironment (TIME) in recurrent GBM, potentially reinvigorating antitumor immune responses at both local and systemic levels and enhancing the infiltration of immune cells into the tumor [5,6].

Recent clinical studies indicate that neoadjuvant PD-1 blockade can improve the survival of GBM patients [7,8]. While this approach shows promise in enhancing immune responses and improving survival outcomes, challenges remain in addressing the immunosuppressive tumor microenvironment [9,10]. Further research is needed to optimize these therapies and explore combination strategies that could enhance their efficacy. Autophagy, a critical intracellular process that facilitates the degradation and recycling of cellular components, plays a vital role in maintaining cellular homeostasis [12]. Treatment with anti-PD-1 antibodies, such as nivolumab, not only enhances the cytotoxic capabilities of T cells but also has the potential to modulate autophagy [11,12]. This modulation could serve as a significant biomarker for treatment response and patient prognosis. Since autophagy might be implicated in drug resistance mechanisms, current research efforts are continuously delving into synergistic treatment approaches that integrate autophagy inhibitors, with a view to enhancing therapeutic outcomes for patients [13].

This study aimed to investigate how neoadjuvant PD-1 blockade impacts immune cell autophagy in the GBM microenvironment. Using single-cell transcriptomics data, we charted immune responses and pinpointed targetable immune cell subtypes regulated by autophagy. Subsequently, the functional characteristics of subtypes regulated by membrane proteins were explored, and potential drugs targeting key immune cell subsets mediated by these proteins were identified. Therefore, our findings provide insights into potential intervention strategies that could enhance the efficacy of anti-PD-1 treatment.

## 2 Methods

### 2.1 Data collection

Single-cell sequencing data from patients with untreated recurrent GBM (GBM.rec) or treated with neoadjuvant anti-PD-1 blockade (GBM.PD1) were obtained from the Gene Expression Omnibus (GEO) database (GSE154795). A total of 12 GBM.rec and 14 GBM.PD1 samples were included in this study. Additionally, we obtained a comprehensive list of genes associated with autophagy from the Human Autophagy Database (HADb; http://www.autophagy.lu/clustering/index.html) [14]. Additionally, we downloaded the GSE4290 dataset from the GEO database to act as an additional bulk validation set.

### 2.2 Cell heterogeneity analysis

To analyze GBM cellular diversity following neoadjuvant PD-1 blockade, we used the *Seurat* R package (v4.1.1) to assess the single-cell sequencing data. We initiated the process by refreshing the foundational dataset using the UpdateSeuratObject function. A shared nearest neighbor graph was constructed using FindNeighbors, paving the way for the discernment of cell subtypes through FindClusters, with a resolution of 0.175 and integrating 30 principal components for nuanced evaluation. Uniform manifold approximation and projection (UMAP) dimensionality reduction was executed using RunUMAP, culminating in the visualization of the cellular subtypes. Differences in gene expression were identified using FindAllMarkers with the following parameters: logfc.threshold = 0.25 and p_val_adj < 0.05. Differentially expressed genes (DEGs) were harnessed as subtype-specific markers, and their identification was further validated against the CellMarker 2.0 database and available literature. The collected information concerning gene expression variances among diverse cell types was visualized using DotPlot and FeaturePlot functions. In addition, we calculated the proportional variances in relevant cell types between the GBM.rec and GBM.PD1 group, thereby gaining a better understanding of the cellular repercussions of this treatment.

Information on a total of 232 genes implicated in autophagy was collected from HADb and the transcriptional profiles of these genes within different cellular groups were evaluated [14]. To quantify the level of autophagy in each cell, we used the single-sample gene set enrichment analysis algorithm implemented in the R package *irGSEA* (v2.1.5) [15]. This approach provided a numerical score indicative of autophagy correlation for every cell analyzed, which was then used to stratify cells into high and low autophagic activity using the median score as threshold.

### 2.3 Construction of a cellular communication network map

Single-cell sequencing data from immune-related cells were used for further analyses. The R package *CellChat* (v1.6.1) was used to identify intercellular communication. First, the overall data were split according to autophagy scores into high and low groups for separate analyses. We constructed a CellChat object using the createCellChat function, set CellChatDB.human as the ligand-receptor interaction database, identified overexpressed genes using the identifyOverExpressedGenes function, and identified overexpressed ligand-receptor interactions using the identifyOverExpressedInteractions function [16]. Subsequently, a protein–protein interaction (PPI) network was constructed using projectData [17]. We calculated communication probabilities and inferred the CellChat network with computeCommunProb. The network was filtered with the filterCommunication function, using a threshold of minimum number of cells = 10. Cell–cell communication at the pathway level was determined using computeCommunProbPathway. The cellular communication network was integrated using aggregateNet. In addition, waterfall maps were designed to highlight the differences in the intensity of the signals received and sent by different cells in each group at the pathway level. Subsequently, the enriched signal pathways between the high and low autophagy groups were counted, and the significance of the observed differences was determined using fish.test.

### 2.4 Functional characteristics of subtypes regulated by membrane proteins

Cells with significant correlations between the GBM.PD1 and GBM.rec groups, as well as between the high and low-autophagy groups were further extracted for follow-up analysis. Lymphocytes subtypes were then divided based on the top 20 principal components with a resolution of 0.1. Based on the subtype markers and CellMarker 2.0 database information (http://bio-bigdata.hrbmu.edu.cn/CellMarker/) [18], we identified the cell subtypes. The identification threshold of characteristic genes between subtypes was logfc.threshold = 0.25 and p_val_adj < 0.05. After the subtype feature genes were identified, gene ontology (GO) and Kyoto Encyclopedia of Genes and Genomes (KEGG) enrichment analyses were performed for each subtype feature gene using the R package *clusterProfiler* (v4.6.2) [19]. The enrichment results were screened for significantly enriched autophagy-related items. Moreover, cells with high expression of membrane proteins in cell communication pathways were considered key subtypes of membrane protein-mediated autophagy. Next, the expression correlation between related subtype-characteristic and autophagy-related genes was calculated. Protein interaction analysis was performed on the characteristic genes of related subtypes using the STRING database (https://cn.string-db.org/) [20]. In addition, we used cell communication analysis to study the interactions between these specific cell subsets and T cells [21], to analyze whether there are different and specific ligand-receptor interactions after treatment with neoadjuvant PD-1 blockade.

### 2.5 Potential drugs that target membrane protein-mediated autotropic key immune cell subtypes

The data of GSE4290 were downloaded, and the characteristic scores of bulk samples were calculated using the R package *GSVA* (v1.34.0) based on the characteristic genes of related subtypes. The samples were divided into high and low groups using the median nodes as the threshold. The R package *limma* (v3.42.2) was used to screen for DEGs between the groups [22], with p_val_adj < 0.05 and |logfc.threshold| > 1. Subsequently, a STRING database was used to construct a PPI network for differential genes and Cytoscape (v3.9.1) was used for visualization [23]. Hub genes were identified based on their degrees using the CytoHubba plugin. Finally, the top 5 hub genes were selected to mine the related drugs according to their interactions, as described in the DGIdb database (https://www.dgidb.org/) [24].

### 2.6 Multiplex immunohistochemistry (mIHC) analysis

Tumor samples of three primary GBM and three paired recurrent GBM were collected (clinical characteristics of patients were shown in Supplementary Table 1), to investigate the expression of AOPE and SQSTM1, as well as the expression of HLA-DRA and CD4 in the two groups. We conducted mIHC based on previous research [25,26]. The following primary antibodies were included: AOPE (dilution: 1:500, Cat No.S0B0703, STARTER), SQSTM1 (dilution: 1:500, Cat No.S0B0586, STARTER), CD4 (dilution: 1:500, Cat No.S0B2179, STARTER) and HLA-DRA (dilution: 1:500, Cat No.17221-1-AP, Proteintech). The sections were counterstained with DAPI for nuclear visualization. Finally, we analyzed their expression using AP-TIME image analysis software (3D Medicines Inc., Shanghai, China).

## 3 Results

### 3.1 Neoadjuvant PD-1 blockade enhances cellular heterogeneity in the GBM microenvironment

Single-cell sequencing data from 12 GBM.rec and 14 GBM.PD1 tissues were analyzed, encompassing a total of 95,497 cells (Supplementary Figure 1A). Eight distinct cell types were identified using marker-based differentiation (Fig. 1A-1C and Supplementary Fig. 1B). Notably, GBM.PD1 tissues exhibited higher infiltration of lymphoid cells and myeloid-lymphocyte-dendritic cells (myeloid-mphage-DC) compared to untreated tissues (Fig. 1D). Subsequent analysis of autophagy in each cell type revealed significant differences in autophagy scores between GBM.PD1 and GBM.rec tissues (Fig. 1E, and Supplementary Fig. 1C and 1D). GBM.PD1 tissues generally exhibited higher autophagy scores in lymphoid cells, proliferating myeloid cells, and myeloid−monocytes, while showing lower scores in microglial cells, myeloid−mphage−DC, tumor/normal brain cells, and stressed/dying cells compared to GBM.rec tissues scores (Fig. 1F). Additionally, both high- and low-autophagy groups displayed significant variations in the frequency of lymphoid and most myeloid cells (Fig. 1G). Lymphoid, myeloid−monocytes, myeloid−lymphocyte−dendritic cells, and proliferating myeloid cells were associated with higher autophagy levels in the GBM.PD1 group, whereas microglial cells were more prevalent among the non-autophagic GBM.rec group (Fig. 1H).

**Fig. 1:**
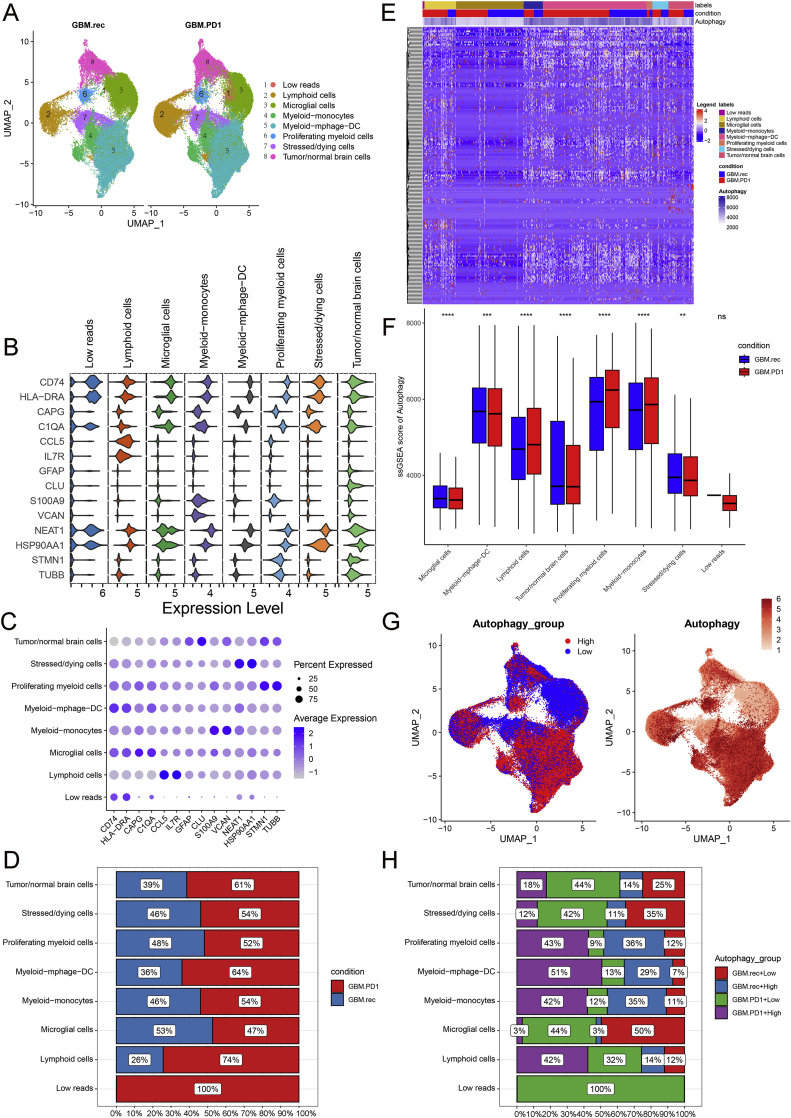
Anti-PD-1 treatment promotes cell heterogeneity within the glioblastoma (GBM) microenvironment. **A.** Uniform manifold approximation and projection (UMAP) of cell distribution in anti-PD-1-treated (GBM.PD1) and untreated (GBM.rec) tissues. **B.** Violin chart showing the expression profile of markers in different cell types. **C.** Bubble maps of markers expressed by different cell types. **D.** Cell proportion analysis among GBM.PD1 and GBM.rec groups. **E.** Expression heatmap of autophagy-related genes. **F.** Differences in autophagy scores between GBM.PD1 and GBM.rec groups. **G.** UMAP display of autophagy status and score. **H.** Cell proportion analysis among integration group by GBM.PD1 and GBM.rec groups, and autophagy score.

### 3.2 High autophagy correlates with specific ligand-receptor interactions in GBM

Analysis of cell communication in the high and low autophagy groups identified 45 and 7 significantly enriched pathways, respectively (Fig. 2A and B, and Supplementary Fig. 2A). Consistent with these findings, the high autophagy group exhibited a greater number and intensity of interactions compared to the low autophagy group (Fig. 2C), suggesting increased cellular activity. Further analysis revealed that 12 of these pathways showed significant differences in the number of interactions between the groups, with related pathways being significantly enriched exclusively in the high-autophagy group (Fig. 2D and Supplementary Fig. 2B). Notably, the HLA-DRA−CD4 signaling pathway emerged as the ligand-receptor pair with the highest contribution (Fig. 2E), with CD4 identified as a key receptor involved in active interaction processes, potentially playing a role in autophagy regulation.

**Fig. 2:**
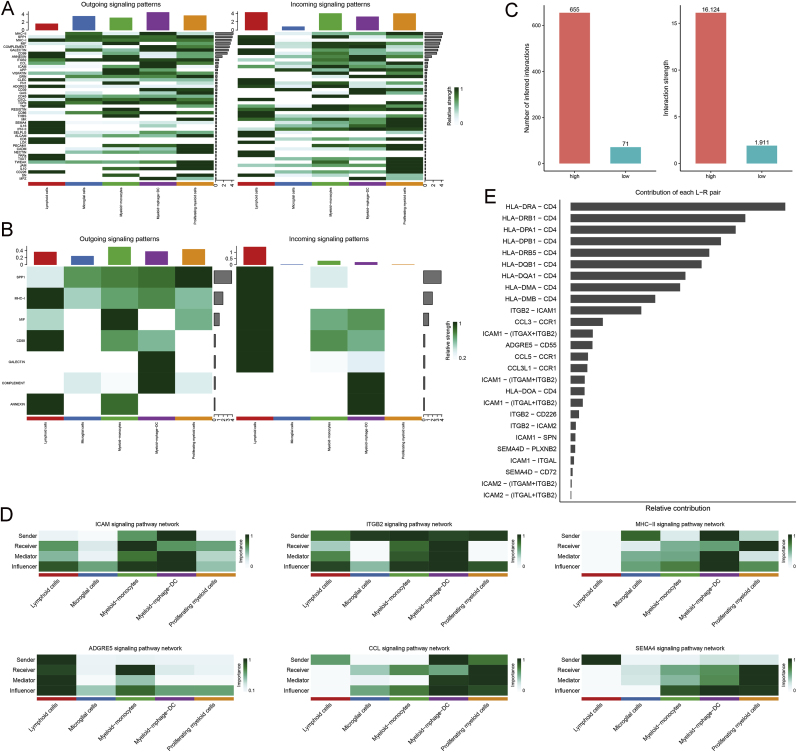
Intercellular communication network in GBM. **A** and **B.** Intensity of outgoing and incoming signaling patterns in the high (A) and low (B) autophagy groups. **C.** Overall statistics on the number and intensity of interactions between the groups. **D.** Top 6 key pathway networks. **E.** Contribution of receptor–ligand interactions in key pathway networks.

### 3.3 L + M cells linked to autophagy in the GBM following neoadjuvant PD-1 blockade

Given the significant differences in lymphoid cells between the GBM.PD1 and GBM.rec group, and between high and low autophagy tissues, further exploration of this subpopulation was warranted. Lymphoid cells were categorized into five distinct subtypes, and their proportions across various tissues were characterized (Fig. 3A–C and Supplementary Fig. 3). Notably, L + M cells, which highly express apolipoprotein E (APOE), were slightly more prevalent in the GBM.PD1 group (Fig. 3D). Autophagy of L + M cells was significantly enhanced (Fig. 3E), and GO and KEGG functional enrichment analyses showed that markers of L + M cells associated with autophagy (Fig. 3F, and Supplementary Fig. 4A–4K), suggesting that L + M cells are a cell type associated with autophagy. We further analyzed the expression of key membrane ligand-receptor pairs in each cell subtype and found that related ligand receptors were predominantly highly expressed in the L + M cells, including HLA-DRA−CD4 (Fig. 3G and Supplementary Fig. 5). Most markers of L + M cells were strongly correlated with autophagy-related genes (Fig. 3H).

**Fig. 3:**
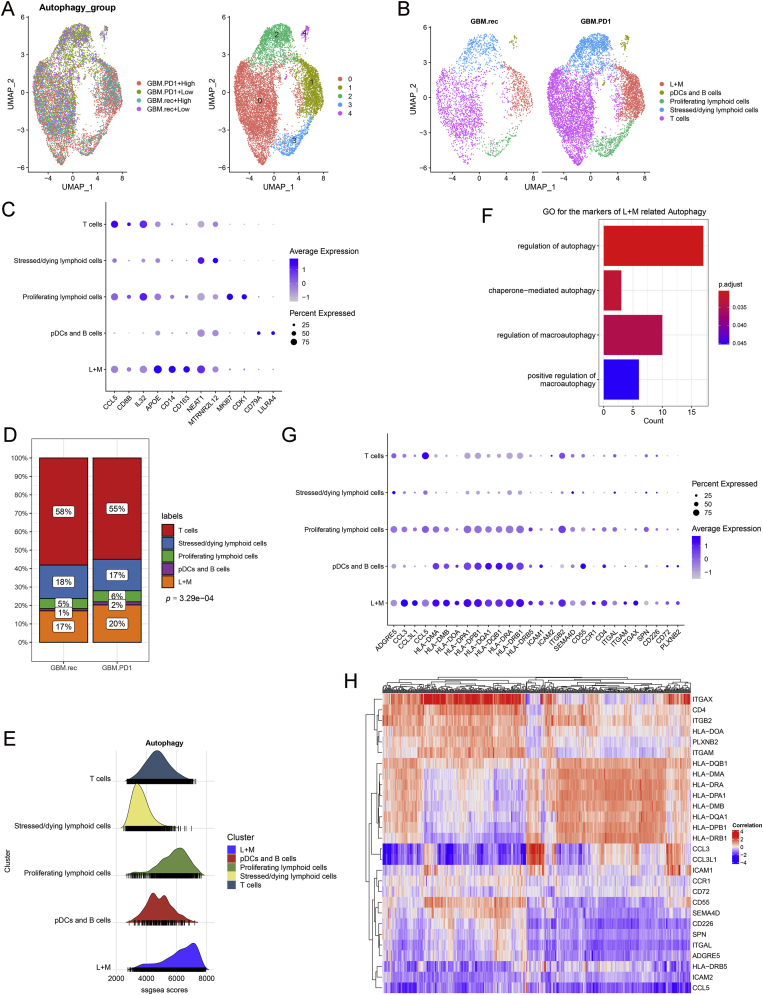
Functional characteristics of cell subtypes regulated by membrane proteins. **A.** Lymphoid cell subtype identification. **B.** Lymphoid cell categorization. **C.** Expression of characteristic genes in each cell subtype. **D.** Frequency of different cell subtypes in the GBM.PD1 and GBM.rec groups. **E.** Autophagy state of different cell subtypes. **F.** Gene ontology enrichment analysis related to autophagy of lymphoid and myeloid (L + M) markers. **G.** Expression of membrane ligand–receptor pairs in each cell subtype. **H.** Correlation between the expression of L + M cell markers and autophagy-related genes.

A protein-protein interaction (PPI) network based on these markers was constructed (Fig. 4A), and significant differences in the expression of six autophagy-related genes (SQSTM1, LAMP2, CTSB, LAMP1, SERPINA1, RGS19) were identified between the GBM.PD1 and GBM.rec groups. Except for LAMP2, the expression of the other five genes was downregulated in the GBM.PD1 group (Fig. 4B and C). Decreased SQSTM1 expression often represents increased autophagy, so these results also suggest that neoadjuvant PD-1 blockade induces the autophagy of L + M cells.

**Fig. 4:**
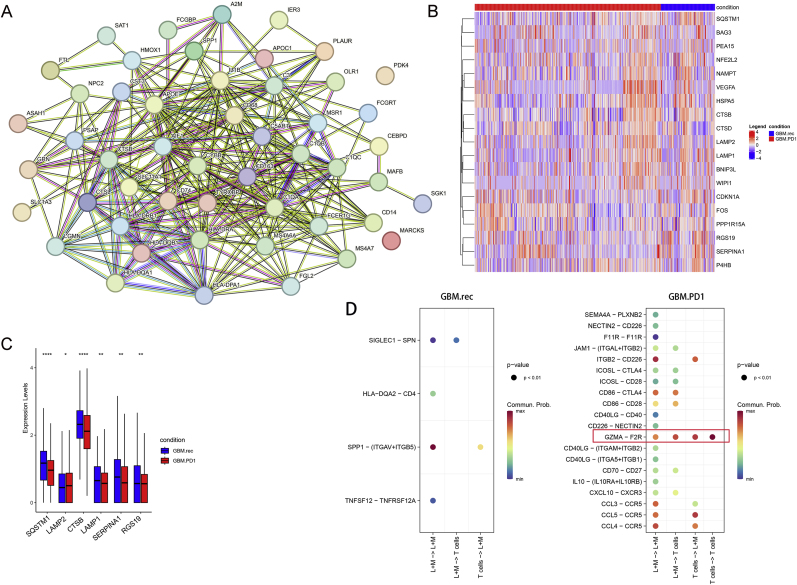
L + M cells as key immune cells related to PD-1 blockade-induced autophagy. **A.** Protein–protein interaction networks for L + M cell markers. **B.** Heatmap of 19 autophagy gene in the GBM.PD1 and GBM.rec groups. **C.** The expression of 6 genes was significantly different between the GBM.PD1 and GBM.rec groups. **D.** Many specific receptor-ligand interactions in the GBM.PD1 group.

To elucidate the interactions between L + M cells and T cells, cell communication analysis was conducted, revealing that L + M cells had significantly more T cell-related interactions in the GBM.PD1 group than in the GBM.rec group, with several specific receptor-ligand interactions observed in the GBM.PD1 group, such as GZMA-F2R interactions (Fig. 4D and Supplementary Fig. 6).

Because APOE is specifically expressed in L + M cells and the change of SQSTM1 expression can represent the strength of autophagy, so we conducted an analysis comparing the expression levels of APOE and SQSTM1 in primary GBM and matched recurrent GBM tissues, to reveal the changes of L + M cells in recurrent GBM. Our findings indicated the expression of APOE was increased in recurrent GBM tissues, and a significant decrease in SQSTM1 expression within APOE + cells in recurrent GBM tissues (Fig. 5A), suggesting an enhancement of autophagy in L + M cells of recurrent GBM. We found earlier that HLA-DRA−CD4 is an important ligand-receptor pairs in the L + M cells, so we compared the expression of HLA-DRA and CD4 in primary and recurrent GBM tissues. Results showed a significant reduction in the expression of HLA-DRA and CD4 in recurrent GBM (Fig. 5B).

**Fig. 5:**
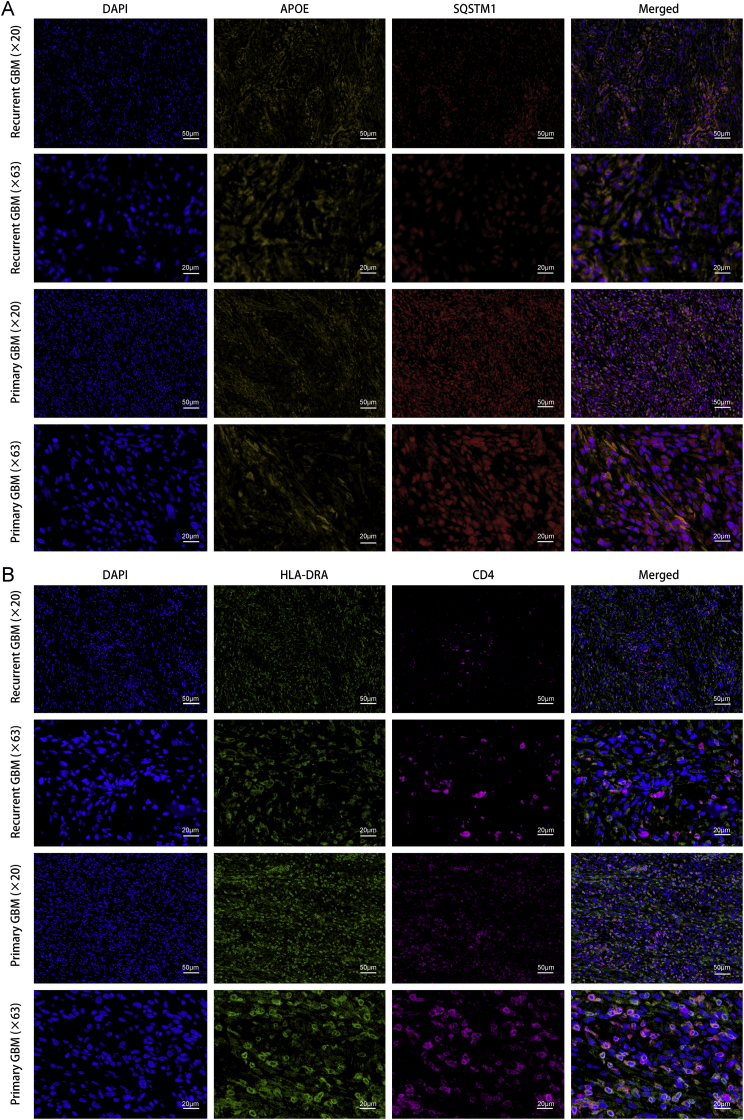
The expression of APOE and SQSTM1, and HLA-DRA and CD4 in primary GBM and matched recurrent GBM. **A.** The expression of APOE and SQSTM1 in primary GBM and matched recurrent GBM. **B.** The expression of HLA-DRA and CD4 in primary GBM and matched recurrent GBM.

### 3.4 Drug screening for L + M cells to enhance the efficacy of neoadjuvant PD-1 blockade in GBM

We carried out drug screening targeting L + M cells in a validation cohort of 77 GBM tissues. Enrichment scores based on L + M cell markers were computed and used to stratify the tissues into high and low enrichment groups, using the median enrichment score as a cutoff. A total of 229 DEGs were identified through this analysis and utilized to construct a PPI network (Fig. 6A). The network encompassed 1518 interactions, and hub genes were identified based on the highest degree of connectivity, with Top 20, Top 10, and Top 5 thresholds being applied (Fig. 6B–D). The PPI analysis highlighted five hub genes with the most significant interaction degrees. Subsequently, we assessed available drugs capable of directly interacting with these hub genes, identifying a total of 73 gene-drug pairs. Of particular interest, the majority of these drugs were found to interact with CXC motif chemokine ligand 8 (CXCL8) and interleukin-6 (IL6). Notably, ribavirin, an antiviral medication, was identified as a potential regulator of both CXCL8 and IL6, implying that it may enhance the efficacy of neoadjuvant PD-1 blockade (Fig. 6E).

**Fig. 6:**
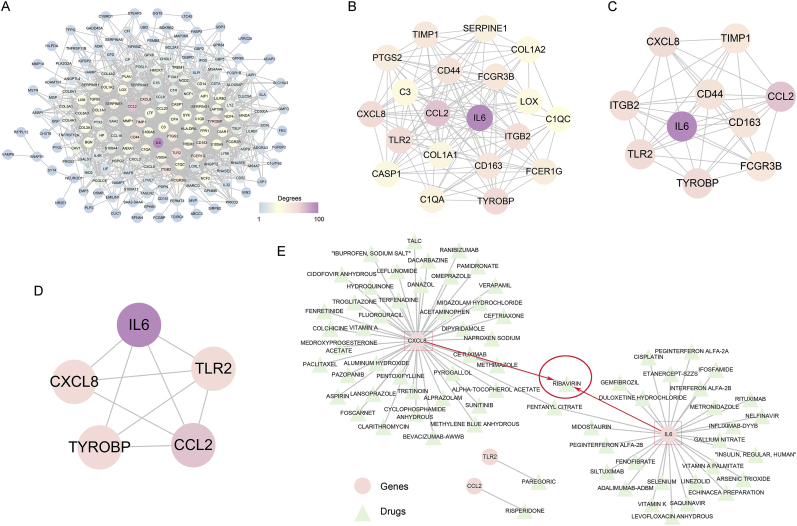
Potential drugs targeting L + M cells. **A–D.** Identification of the PPI network and hub genes based on differentially expressed genes. **E.** Interaction network between hub genes and available drugs.

## 4 Discussion

Neoadjuvant PD-1 blockade has emerged as a promising therapeutic strategy for recurrent GBM. However, existing research has highlighted that anti-PD-1 antibodies can induce autophagy in immune cells, a process that may contribute to treatment resistance [27]. In this study, we investigated the effects of neoadjuvant PD-1 blockade on immune cell autophagy in GBM and made several novel observations. Our results demonstrated a significant increase in the proportion of lymphoid cells exhibiting enhanced autophagy following PD-1 blockade. Importantly, we identified L + M cells - a distinct immune cell subset characterized by dual lymphoid and myeloid signatures - as being strongly associated with autophagy in GBM. To the best of our knowledge, this study is the first to report the regulatory role of L + M cells in autophagy following PD-1 blockade.

L + M cells exhibit high expression of APOE, a molecule with multifaceted roles in the tumor microenvironment, including lipid metabolism, cell signaling, and immune modulation [28]. For example, APOE secreted by prostate tumor cells can bind to neutrophils via TREM2, inducing cell senescence [29]. In pancreatic cancer, TAMs expressing high levels of APOE exert immunosuppressive effects [30]. Notably, APOE peptide-directed chimeric polymersomes have been shown to inhibit GBM growth effectively [31]. Our study revealed increased APOE expression and decreased SQSTM1 expression within APOE + cells in recurrent GBM tissues, indicative of heightened autophagy in L + M cells associated with recurrent GBM. This enhanced autophagy may contribute to tumor recurrence or immune evasion. Furthermore, we observed that increased autophagy in L + M cells was linked to enhanced cellular communication, particularly through the HLA-DRA–CD4 interaction, which emerged as a potentially crucial regulatory ligand-receptor pair. HLA-DRA and CD4 are key immune molecules [32,33]. HLA-DRA, a major histocompatibility complex class II (MHC II) molecule, is essential for presenting extracellular antigens to CD4^+^ T cells, thereby initiating immune responses [34]. HLA-DRA has also been identified as a marker for cancer hotspots and may help predict therapeutic responses to anti-PD-1 immunotherapy in non-small-cell lung cancer [33]. CD4^+^ lymphocytopenia has been implicated in resistance to PD-1 blockade in GBM [35]. In our study, we observed a significant decrease in HLA-DRA and CD4 levels in recurrent GBM and found that L + M cells in the GBM.PD1 group had substantially increased T cell-related interactions compared to the GBM.rec group. Specifically, the GBM.PD1 group exhibited notable enrichment of receptor-ligand interactions, including GZMA-F2R. These findings suggest that L + M cells may modulate immune responses and potentially facilitate immune evasion, warranting further investigation.

It is important to acknowledge that our study has several limitations. First, the analysis was based on retrospective data obtained from the GEO database, which inherently limits our ability to establish causality and may introduce biases related to patient selection and data collection. Additionally, while we identified key interactions and potential therapeutic targets, the functional significance of these findings requires further validation.

Future studies could build on our findings by validating the key mechanisms identified in this study through prospective clinical cohorts or in vitro models. For instance, the association between L + M cell autophagy and HLA-DRA-CD4 interactions could be explored in prospective clinical trials, where patients receiving neoadjuvant PD-1 blockade could be monitored for changes in L + M cell autophagy and clinical outcomes. Furthermore, in vitro models, such as PD-1-treated GBM cell lines or co-culture systems incorporating L + M cells, could provide valuable insights into the functional role of these interactions and their impact on immune responses and tumor progression.

In our search for therapeutic targets, we identified that modulating CXCL8 or IL6 could enhance the efficacy of neoadjuvant PD-1 blockade by specifically targeting L + M cells. CXCL8 is primarily recognized for attracting and activating immune cells such as neutrophils, monocytes, and T cells, and is implicated in various tumor progressions. For instance, CXCL8 supports the mesenchymal state of GBM stem cells and promotes M2-like 10.13039/100026873TAM polarization; its inhibition has been shown to suppress GBM growth [36]. CXCL8 inhibitors have also been reported to enhance antitumor immunity [37]. IL6, produced by multiple cell types, including activated T cells, macrophages, and fibroblasts, plays a role in tumor immunity regulation. Elevated IL6 levels have been linked to glioma growth, with hypoxia-induced IL6 secretion enhancing GBM autophagy; hence, targeted inhibition by tocilizumab can prevent GBM progression and autophagy [38]. Drug-gene interaction network analysis identified ribavirin, widely recognized as an antiviral agent for hepatitis C virus therapy, as a potential therapeutic agent for targeting CXCL8 and IL6. ribavirin shows promise for repurposing in cancer treatment. It has exhibited significant therapeutic potential across a range of cancers, such as acute myeloid leukemia, nasopharyngeal carcinoma, and lung adenocarcinoma [39,40]. Furthermore, ribavirin has been suggested to inhibit GBM growth and enhance the effectiveness of chemo-radiotherapy [41,42], reduce chemo-resistant tumor growth of triple negative breast cancer [43], and promote the expression of PD-L1 in cancer cells without impairing the efficacy of immunotherapy [44].

In conclusion, this study underscores L + M cells as critical immune components associated with autophagy induced by neoadjuvant PD-1 blockade. Interactions between HLA-DRA and CD4, as well as GZMA and F2R, are important for modulating immune responses. Furthermore, ribavirin, which targets CXCL8 and IL6, presents a promising strategy to enhance the efficacy of anti-PD-1 therapy. Our findings not only provide novel insights into the immune mechanisms underlying neoadjuvant PD-1 blockade but also offer potential therapeutic avenues for improving outcomes in GBM patients. Future studies should further investigate the functional roles of L + M cells and validate the clinical efficacy of ribavirin in combination with PD-1 blockade.

## CRediT authorship contribution statement

**Zixue Xuan:** Writing – original draft, Funding acquisition, Formal analysis, Data curation, Conceptualization. **Kai Wang:** Writing – original draft, Data curation. **Qingxia Zhu:** Writing – review & editing. **Ting Sun:** Formal analysis. **Jinying Jiang:** Data curation. **Zhongxiu Wu:** Writing – review & editing. **Shuilian Zheng:** Writing – review & editing, Methodology. **Hongying Zhao:** Supervision, Funding acquisition, Data curation.

## Availability of data and materials

All data generated or analyzed during this study were included in this article. The datasets used and analyzed in the current study are available from the corresponding author upon reasonable request.

## Ethical approval and consent to participate

The study was approved by the Ethics Committee of the Zhejiang Provincial People’s Hospital (QT2022301).

## Consent for publication

Not applicable.

## Financial Support and sponsorship

This work was supported by Medical Science and Technology Project of Zhejiang Province (No.2021KY463, 2022KY607), Bijie City personalized medicine research talent team project.

## Declaration of competing interest

The authors declare the following financial interests/personal relationships which may be considered as potential competing interests: Hongying Zhao reports financial support was provided by Zhejiang Provincial People’s Hospital. Zixue Xuan reports financial support was provided by Zhejiang Provincial People’s Hospital. If there are other authors, they declare that they have no known competing financial interests or personal relationships that could have appeared to influence the work reported in this paper.

## Figures and Tables

**figs1:**
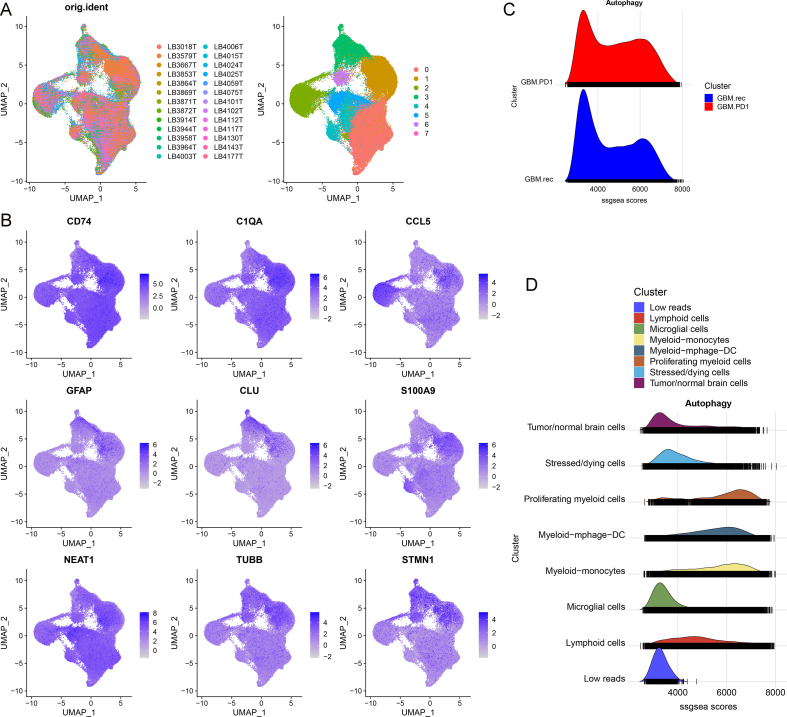


**figs2:**
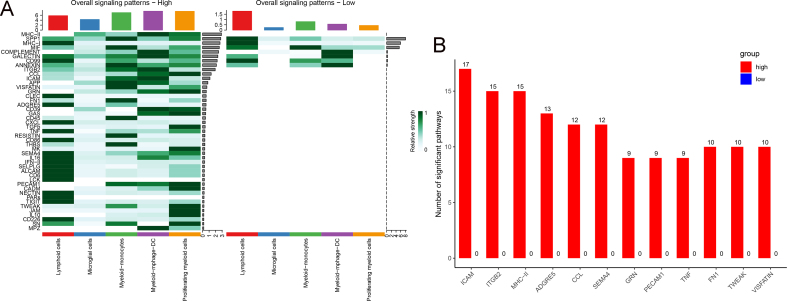


**figs3:**
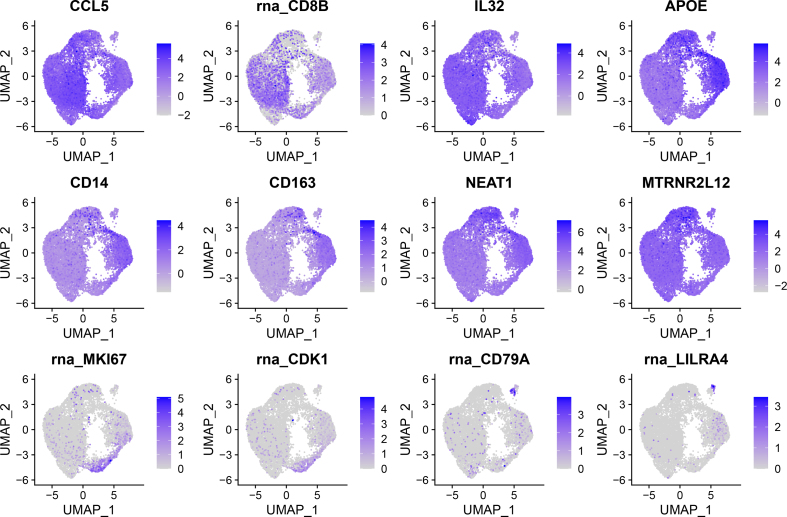


**figs4:**
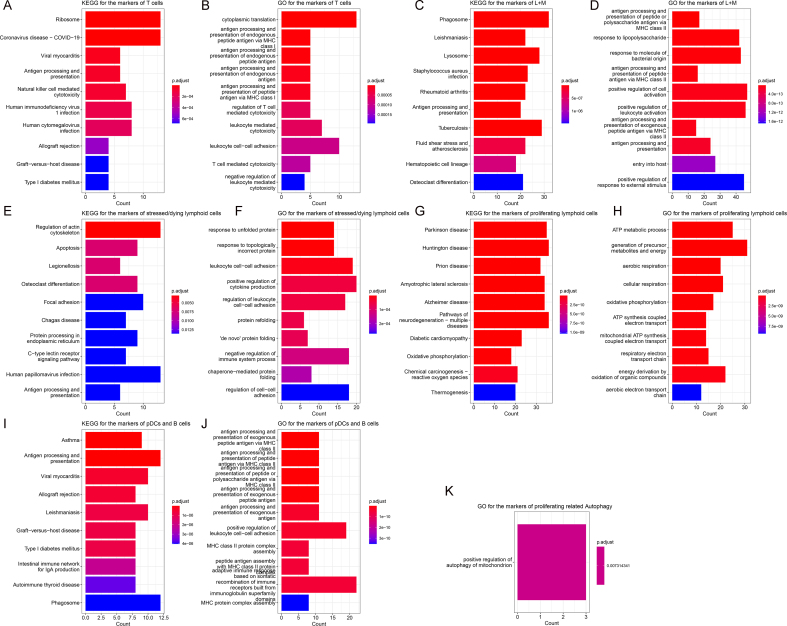


**figs5:**
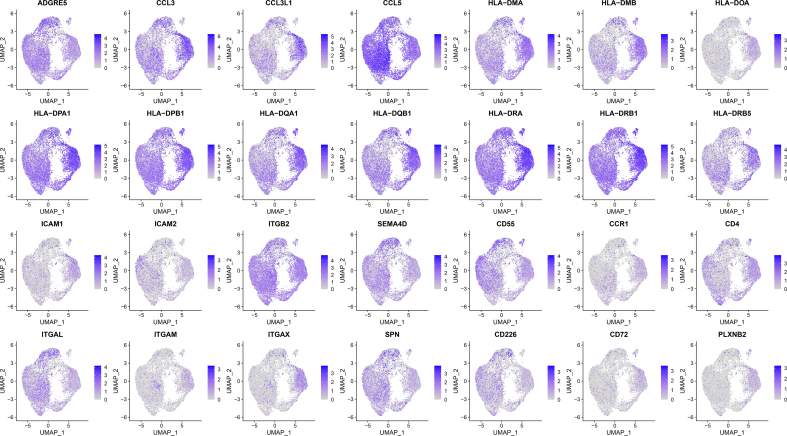


**figs6:**
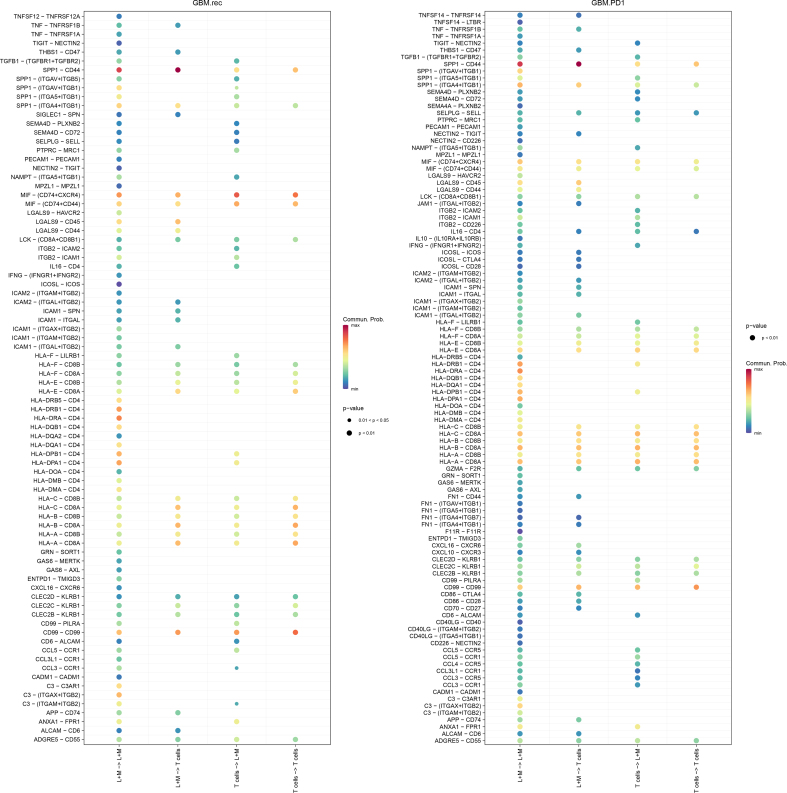


## Data Availability

Data will be made available on request.

## References

[1] Read R.D., Tapp Z.M., Rajappa P., Hambardzumyan D. (2024). Glioblastoma microenvironment-from biology to therapy. Genes Dev..

[2] Omuro A., Brandes A.A., Carpentier A.F., Idbaih A., Reardon D.A., Cloughesy T., Sumrall A., Baehring J., van den Bent M., Bähr O., Lombardi G., Mulholland P., Tabatabai G., Lassen U., Sepulveda J.M., Khasraw M., Vauleon E., Muragaki Y., Di Giacomo A.M., Butowski N., Roth P., Qian X., Fu A.Z., Liu Y., Potter V., Chalamandaris A.G., Tatsuoka K., Lim M., Weller M. (2023). Radiotherapy combined with nivolumab or temozolomide for newly diagnosed glioblastoma with unmethylated MGMT promoter: an international randomized phase III trial. Neuro Oncol..

[3] Lim M., Weller M., Idbaih A., Steinbach J., Finocchiaro G., Raval R.R., Ansstas G., Baehring J., Taylor J.W., Honnorat J., Petrecca K., De Vos F., Wick A., Sumrall A., Sahebjam S., Mellinghoff I.K., Kinoshita M., Roberts M., Slepetis R., Warad D., Leung D., Lee M., Reardon D.A., Omuro A. (2022). Phase III trial of chemoradiotherapy with temozolomide plus nivolumab or placebo for newly diagnosed glioblastoma with methylated MGMT promoter. Neuro Oncol..

[4] Reardon D.A., Brandes A.A., Omuro A., Mulholland P., Lim M., Wick A., Baehring J., Ahluwalia M.S., Roth P., Bähr O., Phuphanich S., Sepulveda J.M., De Souza P., Sahebjam S., Carleton M., Tatsuoka K., Taitt C., Zwirtes R., Sampson J., Weller M. (2020). Effect of nivolumab vs bevacizumab in patients with recurrent glioblastoma: the CheckMate 143 phase 3 randomized clinical trial. JAMA Oncol..

[5] Park J.H., Kang I., Lee H.K. (2022). The immune landscape of high-grade brain tumor after treatment with immune checkpoint blockade. Front. Immunol..

[6] Simonds E.F., Lu E.D., Badillo O., Karimi S., Liu E.V., Tamaki W., Rancan C., Downey K.M., Stultz J., Sinha M., McHenry L.K., Nasholm N.M., Chuntova P., Sundström A., Genoud V., Shahani S.A., Wang L.D., Brown C.E., Walker P.R., Swartling F.J., Fong L., Okada H., Weiss W.A., Hellström M. (2021). Deep immune profiling reveals targetable mechanisms of immune evasion in immune checkpoint inhibitor-refractory glioblastoma. J. Immunother. Cancer.

[7] Schalper K.A., Rodriguez-Ruiz M.E., Diez-Valle R., López-Janeiro A., Porciuncula A., Idoate M.A., Inogés S., de Andrea C., López-Diaz de Cerio A., Tejada S., Berraondo P., Villarroel-Espindola F., Choi J., Gúrpide A., Giraldez M., Goicoechea I., Gallego Perez-Larraya J., Sanmamed M.F., Perez-Gracia J.L., Melero I. (2019). Neoadjuvant nivolumab modifies the tumor immune microenvironment in resectable glioblastoma. Nat Med.

[8] Cloughesy T.F., Mochizuki A.Y., Orpilla J.R., Hugo W., Lee A.H., Davidson T.B., Wang A.C., Ellingson B.M., Rytlewski J.A., Sanders C.M., Kawaguchi E.S., Du L., Li G., Yong W.H., Gaffey S.C., Cohen A.L., Mellinghoff I.K., Lee E.Q., Reardon D.A., O'Brien B.J., Butowski N.A., Nghiemphu P.L., Clarke J.L., Arrillaga-Romany I.C., Colman H., Kaley T.J., de Groot J.F., Liau L.M., Wen P.Y., Prins R.M. (2019). Neoadjuvant anti-PD-1 immunotherapy promotes a survival benefit with intratumoral and systemic immune responses in recurrent glioblastoma. Nat Med.

[9] Sun L., Lai T.J., Prins R.M. (2021). Is there a role for neoadjuvant anti-PD-1 therapies in glioma?. Curr. Opin. Neurol..

[10] Lee A.H., Sun L., Mochizuki A.Y., Reynoso J.G., Orpilla J., Chow F., Kienzler J.C., Everson R.G., Nathanson D.A., Bensinger S.J., Liau L.M., Cloughesy T., Hugo W., Prins R.M. (2021). Neoadjuvant PD-1 blockade induces T cell and cDC1 activation but fails to overcome the immunosuppressive tumor associated macrophages in recurrent glioblastoma. Nat. Commun..

[11] Yu Q., Ding J., Li S., Li Y. (2024). Autophagy in cancer immunotherapy: perspective on immune evasion and cell death interactions. Cancer Lett..

[12] Jiang T., Chen X., Ren X., Yang J.M., Cheng Y. (2021). Emerging role of autophagy in anti-tumor immunity: implications for the modulation of immunotherapy resistance. Drug Resist. Updates : reviews and commentaries in antimicrobial and anticancer chemotherapy.

[13] Ishimwe N., Zhang W., Qian J., Zhang Y., Wen L. (2020). Autophagy regulation as a promising approach for improving cancer immunotherapy. Cancer Lett..

[14] Wang Y., Huang S., Zhang Y., Cheng Y., Dai L., Gao W., Feng Z., Tao J., Zhang Y. (2023). Construction and validation of a prognostic model based on autophagy-related genes for hepatocellular carcinoma in the Asian population. BMC Genom..

[15] Fan C., Chen F., Chen Y., Huang L., Wang M., Liu Y., Wang Y., Guo H., Zheng N., Liu Y., Wang H., Ma L. (2024). irGSEA: the integration of single-cell rank-based gene set enrichment analysis. Brief Bioinform.

[16] Lin J., Cai Y., Wang Z., Ma Y., Pan J., Liu Y., Zhao Z. (2022). Novel biomarkers predict prognosis and drug-induced neuroendocrine differentiation in patients with prostate cancer. Front. Endocrinol..

[17] Sun Z., Yun Z., Lin J., Sun X., Wang Q., Duan J., Li C., Zhang X., Xu S., Wang Z., Xiong X., Yao K. (2024). Comprehensive mendelian randomization analysis of plasma proteomics to identify new therapeutic targets for the treatment of coronary heart disease and myocardial infarction. J. Transl. Med..

[18] Hu C., Li T., Xu Y., Zhang X., Li F., Bai J., Chen J., Jiang W., Yang K., Ou Q., Li X., Wang P., Zhang Y. (2023). CellMarker 2.0: an updated database of manually curated cell markers in human/mouse and web tools based on scRNA-seq data. Nucleic Acids Res..

[19] Lee S., Chen D., Park M., Kim S., Choi Y.J., Moon S.J., Shin D.M., Lee J.H., Kim E. (2022). Single-cell RNA sequencing analysis of human dental pulp stem cell and human periodontal ligament stem cell. J. Endod..

[20] Szklarczyk D., Kirsch R., Koutrouli M., Nastou K., Mehryary F., Hachilif R., Gable A.L., Fang T., Doncheva N.T., Pyysalo S., Bork P., Jensen L.J., von Mering C. (2023). The STRING database in 2023: protein-protein association networks and functional enrichment analyses for any sequenced genome of interest. Nucleic Acids Res..

[21] Cheng C., Chen W., Jin H., Chen X. (2023). A review of single-cell RNA-seq annotation, integration, and cell-cell communication. Cells.

[22] Ritchie M.E., Phipson B., Wu D., Hu Y., Law C.W., Shi W., Smyth G.K. (2015). limma powers differential expression analyses for RNA-sequencing and microarray studies. Nucleic Acids Res..

[23] Shao F.L., Liu Q.Q., Wang S. (2021). Identify potential miRNA-mRNA regulatory networks contributing to high-risk neuroblastoma. Invest. N. Drugs.

[24] Cannon M., Stevenson J., Stahl K., Basu R., Coffman A., Kiwala S., McMichael J.F., Kuzma K., Morrissey D., Cotto K., Mardis E.R., Griffith O.L., Griffith M., Wagner A.H. (2024). DGIdb 5.0: rebuilding the drug-gene interaction database for precision medicine and drug discovery platforms. Nucleic Acids Res..

[25] Xuan Z., Liu L., Zhang G., Zheng X., Jiang J., Wang K., Huang P. (2023). Novel cell subtypes of SPP1 + S100P+, MS4A1-SPP1 + S100P+ were key subpopulations in intrahepatic cholangiocarcinoma. Biochimica et biophysica acta. General subjects.

[26] Xuan Z., Fang L., Zhang G., Zhang X., Jiang J., Wang K., Huang P. (2023). The heterogeneity of tumour-associated macrophages contributes to the recurrence and outcomes of glioblastoma patients. J. Mol. Neurosci..

[27] Yamamoto K., Venida A., Yano J., Biancur D.E., Kakiuchi M., Gupta S., Sohn A.S.W., Mukhopadhyay S., Lin E.Y., Parker S.J., Banh R.S., Paulo J.A., Wen K.W., Debnath J., Kim G.E., Mancias J.D., Fearon D.T., Perera R.M., Kimmelman A.C. (2020). Autophagy promotes immune evasion of pancreatic cancer by degrading MHC-I. Nature.

[28] INVALID CITATION !!! [28,29]).

[29] Bancaro N., Calì B., Troiani M., Elia A.R., Arzola R.A., Attanasio G., Lai P., Crespo M., Gurel B., Pereira R., Guo C., Mosole S., Brina D., D'Ambrosio M., Pasquini E., Spataro C., Zagato E., Rinaldi A., Pedotti M., Di Lascio S., Meani F., Montopoli M., Ferrari M., Gallina A., Varani L., Pereira Mestre R., Bolis M., Gillessen Sommer S., de Bono J., Calcinotto A., Alimonti A. (2023). Apolipoprotein E induces pathogenic senescent-like myeloid cells in prostate cancer. Cancer Cell.

[30] Kemp S.B., Carpenter E.S., Steele N.G., Donahue K.L., Nwosu Z.C., Pacheco A., Velez-Delgado A., Menjivar R.E., Lima F., The S., Espinoza C.E., Brown K., Long D., Lyssiotis C.A., Rao A., Zhang Y., Pasca di Magliano M., Crawford H.C. (2021). Apolipoprotein E promotes immune suppression in pancreatic cancer through NF-κB-Mediated production of CXCL1. Cancer Res..

[31] Jiang Y., Zhang J., Meng F., Zhong Z. (2018). Apolipoprotein E peptide-directed chimeric polymersomes mediate an ultrahigh-efficiency targeted protein therapy for glioblastoma. ACS Nano.

[32] Liu D., Hofman P. (2022). Expression of NOTCH1, NOTCH4, HLA-DMA and HLA-DRA is synergistically associated with T cell exclusion, immune checkpoint blockade efficacy and recurrence risk in ER-negative breast cancer. Cell. Oncol..

[33] Mei J., Jiang G., Chen Y., Xu Y., Wan Y., Chen R., Liu F., Mao W., Zheng M., Xu J. (2022). HLA class II molecule HLA-DRA identifies immuno-hot tumors and predicts the therapeutic response to anti-PD-1 immunotherapy in NSCLC. BMC Cancer.

[34] Wang B., Liu Y., Xiong F., Wang C. (2024). Improved immunotherapy outcomes via cuproptosis upregulation of HLA-DRA expression: promoting the aggregation of CD4(+) and CD8(+)T lymphocytes in clear cell renal cell carcinoma. Pharmaceuticals.

[35] Khan S.M., Desai R., Coxon A., Livingstone A., Dunn G.P., Petti A., Johanns T.M. (2022). Impact of CD4 T cells on intratumoral CD8 T-cell exhaustion and responsiveness to PD-1 blockade therapy in mouse brain tumors. J. Immunother. Cancer.

[36] Yuan W., Zhang Q., Gu D., Lu C., Dixit D., Gimple R.C., Gao Y., Gao J., Li D., Shan D., Hu L., Li L., Li Y., Ci S., You H., Yan L., Chen K., Zhao N., Xu C., Lan J., Liu D., Zhang J., Shi Z., Wu Q., Yang K., Zhao L., Qiu Z., Lv D., Gao W., Yang H., Lin F., Wang Q., Man J., Li C., Tao W., Agnihotri S., Qian X., Mack S.C., Zhang N., You Y., Rich J.N., Sun G., Wang X. (2023). Dual role of CXCL8 in maintaining the mesenchymal state of glioblastoma stem cells and M2-Like tumor-associated macrophages. Clin. Cancer Res..

[37] Lin C., He H., Liu H., Li R., Chen Y., Qi Y., Jiang Q., Chen L., Zhang P., Zhang H., Li H., Zhang W., Sun Y., Xu J. (2019). Tumour-associated macrophages-derived CXCL8 determines immune evasion through autonomous PD-L1 expression in gastric cancer. Gut.

[38] Xue H., Yuan G., Guo X., Liu Q., Zhang J., Gao X., Guo X., Xu S., Li T., Shao Q., Yan S., Li G. (2016). A novel tumor-promoting mechanism of IL6 and the therapeutic efficacy of tocilizumab: hypoxia-Induced IL6 is a potent autophagy initiator in glioblastoma via the p-STAT3-MIR155-3p-CREBRF pathway. Autophagy.

[39] Borden K.L., Culjkovic-Kraljacic B. (2010). Ribavirin as an anti-cancer therapy: acute myeloid leukemia and beyond?. Leuk. Lymphoma.

[40] Paudel K.R., Singh M., De Rubis G., Kumbhar P., Mehndiratta S., Kokkinis S., El-Sherkawi T., Gupta G., Singh S.K., Malik M.Z., Mohammed Y., Oliver B.G., Disouza J., Patravale V., Hansbro P.M., Dua K. (2024). Computational and biological approaches in repurposing ribavirin for lung cancer treatment: unveiling antitumorigenic strategies. Life Sci..

[41] Volpin F., Casaos J., Sesen J., Mangraviti A., Choi J., Gorelick N., Frikeche J., Lott T., Felder R., Scotland S.J., Eisinger-Mathason T.S.K., Brem H., Tyler B., Skuli N. (2017). Use of an anti-viral drug, ribavirin, as an anti-glioblastoma therapeutic. Oncogene.

[42] Ochiai Y., Sano E., Okamoto Y., Yoshimura S., Makita K., Yamamuro S., Ohta T., Ogino A., Tadakuma H., Ueda T., Nakayama T., Hara H., Yoshino A., Katayama Y. (2018). Efficacy of ribavirin against malignant glioma cell lines: follow-up study. Oncol. Rep..

[43] da Silva Fernandes T., Gillard B.M., Dai T., Martin J.C., Chaudhry K.A., Dugas S.M., Fisher A.A., Sharma P., Wu R., Attwood K.M., Dasgupta S., Takabe K., Rosario S.R., Bianchi-Smiraglia A. (2025). Inosine monophosphate dehydrogenase 2 (IMPDH2) modulates response to therapy and chemo-resistance in triple negative breast cancer. Sci. Rep..

[44] Zheng M.M., Li J.Y., Guo H.J., Zhang J., Wang L.S., Jiang K.F., Wu H.H., He Q.J., Ding L., Yang B. (2025). IMPDH inhibitors upregulate PD-L1 in cancer cells without impairing immune checkpoint inhibitor efficacy. Acta Pharmacol. Sin..

